# Proton-conducting Micro-solid Oxide Fuel Cells with Improved Cathode Reactions by a Nanoscale Thin Film Gadolinium-doped Ceria Interlayer

**DOI:** 10.1038/srep22369

**Published:** 2016-02-29

**Authors:** Yong Li, Shijie Wang, Pei-Chen Su

**Affiliations:** 1 School of Mechanical and Aerospace Engineering, Nanyang Technological University, 50 Nanyang Avenue, Singapore 639798; 2 Institute of Materials Research and Engineering, Agency for Science, Technology and Research (A*STAR), 2 Fusionopolis Way, Singapore 138634

## Abstract

An 8 nm-thick gadolinium-doped ceria (GDC) layer was inserted as a cathodic interlayer between the nanoscale proton-conducting yttrium-doped barium zirconate (BZY) electrolyte and the porous platinum cathode of a micro-solid oxide fuel cell (μ-SOFC), which has effectively improved the cathode reaction kinetics and rendered high cell power density. The addition of the GDC interlayer significantly reduced the cathodic activation loss and increased the peak power density of the μ-SOFC by 33% at 400 °C. The peak power density reached 445 mW/cm^2^ at 425 °C, which is the highest among the reported μ-SOFCs using proton-conducting electrolytes. The impressive performance was attributed to the mixed protonic and oxygen ionic conducting properties of the nano-granular GDC, and also to the high densities of grain boundaries and lattice defects in GDC interlayer that favored the oxygen incorporation and transportation during the oxygen reduction reaction (ORR) and the water evolution reaction at cathode.

Micro-solid oxide fuel cells (μ-SOFCs) using nanoscale thin film electrolytes have shown a great promise as portable power sources because of their high performance at drastically reduced operating temperatures^1^. By minimizing the electrolyte thickness from tens of micrometers scale down to sub-micrometer scale, Ohmic resistance of conventional oxygen ion-conducting electrolytes such as yttria-stabilized zirconia (YSZ) decrease proportionally with thickness, which enable the high cell performance at temperatures lower than 500 °C^2^,^3^,^4^,^5^,^6^,^7^. As Ohmic resistance is minimized, the most rate-limiting process among the entire cell reactions is shifted to the cathode polarization, since the thermally-driven oxygen reduction reaction (ORR) kinetics at the cathode becomes much more sluggish at such low temperature range^8^. Therefore, improving the kinetics of cathodic reactions or the selection of catalytically more active cathode materials is currently the most critical issue in further enhancing the performance of such promising devices.

Among reported μ-SOFCs, quite a few works have shown impressive performance using oxygen ion-conducting electrolytes such as YSZ, gadolinium-doped ceria (GDC), or with multiple-layer configurations like GDC/YSZ bilayer electrolyte^2^,^5^. However, for μ-SOFCs operating at their targeted temperature regime, which is usually below 500 °C, proton-conducting oxides can be more suitable choices as electrolyte materials since they usually possess better ionic conductivity than oxygen-ion conductors at low temperature due to lower activation energy of proton conduction^9^. It should be expected that the already impressive performance of μ-SOFCs reported can be further improved if the oxygen ion-conducting electrolyte is replaced with proton-conducting electrolyte while remaining the other cell components such as porous metal catalytic electrodes unchanged. Nevertheless, to date, among the limited number of reports on μ-SOFCs using the most common proton-conducting electrolytes (μ-H-SOFCs hereafter) like yttrium-doped barium zirconate (BZY), the peak power densities reported were still much lower than those using zirconia- or ceria-based oxygen ion-conducting electrolytes (μ-O-SOFCs hereafter). As summarized in Table 1, the highest peak power densities attainable for μ-H-SOFCs were only 140 mW/cm^2^ at 400 °C^10^ and 186 mW/cm^2^ at 450 °C^11^. For the reported high performance of μ-O-SOFCs, the highest peak power densities reported were closer to or over 1 W/cm^2^ at 450 °C^4^,^5^,^6^.

The lower performance of μ-H-SOFCs may originate from the poor cathode configuration that provides insufficient reaction sites on such type of nano thin film fuel cells. Figure 1a shows the typical cathode/electrolyte/anode cross-sectional schematics of the μ-H-SOFC, which has a nano thin film BZY electrolyte sandwiched between two porous thin film Pt electrodes. Unlike conventional SOFCs, the cathode/electrolyte interface of a μ-SOFC is only connected two-dimensionally, which means the triple phase boundary (TPB) for the ORR is only limited to the planar interface between Pt cathode and oxide electrolyte. When the electrolyte is changed from an oxygen ion conductor to a proton conductor, in addition to the existing ORR, the water evolution reaction also shifts from anode to cathode, making the complex cathode reactions even more complicated. In BZY μ-H-SOFCs, the reduced oxygen ion may only meet the proton transported from the anode through BZY near the TPB lines at the interface between Pt cathode and BZY (red line in Fig. 1a), and these are the likely places where the water evolution reaction occurs. Therefore, it is expected that the performance of μ-H-SOFCs has been limited by the confined reaction zone that resulted in high cathodic polarization resistance. In this regard, modifying the interface between Pt and BZY to allow the cathodic reactions to take place over an extended zone is expected to effectively improve the cathode kinetics and increase the cell power performance. Among studies for regular proton-conducting electrolyte SOFCs (H-SOFCs) operated at intermediate temperature range (500 to 650 °C), the search of suitable cathode materials to accommodate the complex cathode reactions is also a prevailing topic^12^,^13^,^14^,^15^. One of the most promising categories of cathode is triple-conducting materials, which are materials having simultaneous electronic, oxygen ionic, and protonic conduction properties^16^. A triple-conducting cathode provides more reaction sites for both the ORR and water evolution reaction to take place that is believed to effectively decrease the cathode polarization resistance, and the reported cell performances using such cathode material are indeed impressive^13^. To apply such concept to μ-H-SOFCs, adding an interlayer between the cathode and electrolyte, or the so-called bi-layered electrolyte, can be an effective method.

For μ-O-SOFCs, the concept of bi-layered electrolytes or the cathode/electrolyte interface modifications have been demonstrated to effectively improve the cathode kinetics. A catalytically superior material for ORR, such as doped-ceria^2^,^5^,^17^, can be inserted between the porous metallic cathode and the main electrolyte to serve as a good cation diffusion barrier^18^ and improve the ORR reaction kinetics^19^,^20^. Results from quantum mechanical simulation also showed very low oxygen incorporation energetics, of 0.07 eV for doped ceria at cathode/electrolyte interface, which is much lower than the 0.38 eV of YSZ^21^.

To apply the bi-layer electrolyte concept to μ-H-SOFCs for better cathode reaction kinetics, GDC can still be a good choice to accommodate the complex cathode reactions. The Ga doping in ceria gives the higher oxygen diffusion coefficient than ceria doped with other dopants such as Y, Sm, and La^22^. More importantly, evidences of proton conduction at temperature lower than 400 °C were also reported^23^,^24^,^25^,^26^. The mixed oxygen ion and proton-conducting property allows both the oxygen ions and protons to transfer into the GDC layer, and therefore it is likely to extend the water formation and evolution zone from the confined TPB lines (Fig. 1a) into part of or the entire GDC layer (Fig. 1b).

Therefore, the purpose of this work is to demonstrate the concept of adding a GDC interlayer which is catalytically more active and is a mixed oxygen ionic and protonic conductor, in order to improve the chronically poor performance of μ-H-SOFCs. An 8 nm-thick GDC layer was deposited on top of BZY proton-conducting electrolyte to serve as a cathode interlayer for μ-H-SOFCs. Cathode performance and fuel cell power density in the silicon-based μ-H-SOFC were significantly improved with the addition of GDC interlayer. The electrochemical impedance and fuel cell performance of the fabricated μ-H-SOFCs with and without the GDC interlayer were characterized to understand the effect of such cathode interlayer on the cathode kinetics behavior.

## Results

### Microstructure of the GDC/BZY Electrolyte

Figure 2 shows the XRD patterns of BZY electrolyte with and without GDC cathodic interlayer. The BZY electrolyte layer has a polycrystalline structure with a preferred orientation of (011). All of the peaks were indexed to the standard BZY PDF card 96-720-2180, which indicates well-crystallized BZY film at the deposition temperature of 800 °C. In the case electrolyte with GDC interlayer, additional GDC peaks were clearly observed and well-matched with the standard GDC pattern of PDF 00-046-0508. The relatively low intensity of GDC peaks was due to the much smaller thickness of GDC interlayer (8 nm) than BZY (75 nm). No additional phase was observed from the bi-layer electrolyte, which means there is no reaction between BZY and GDC during the deposition at 800 °C.

The FESEM cross-sectional images of both the μ-H-SOFCs having the BZY electrolytes with and without GDC interlayer are shown in Fig. 3. The dense BZY electrolytes in both cases were 75 nm in thickness and sandwiched between the porous Pt cathode and anode of 100 nm and 60 nm in thickness, respectively.

Cross-sectional TEM characterizations were performed for GDC/BZY bi-layer electrolyte to investigate the crystallinity and the microstructures (Fig. 4). Both of the fully crystallized BZY electrolyte and GDC interlayer showed columnar grains with vertical grain boundaries parallel to the ion transportation direction (Fig. 4a), which can minimize the cross grain boundary resistance during proton conduction^27^. The thickness of BZY electrolyte and GDC interlayer were confirmed to be 75 nm and 8 nm, respectively (Fig. 4b). No additional phase was visible between the BZY and GDC layers, which suggests good chemical compatibility and stability between these two layers at the deposition temperature of 800 °C, and this is in agreement with the XRD results. Therefore, no reaction is expected to occur during the μ-H-SOFC operation because of the much lower operating temperature (below 500 °C) than the deposition temperature. Although a large mismatch of lattice constant exists between BZY (0.42 nm) and GDC (0.54 nm), the grain boundaries of the GDC interlayer were aligned to the grain boundaries of the underneath BZY during the grain growth from PLD deposition. The grain alignment introduced a compressive stress to the GDC interlayer, which resulted in a high density of dislocations and lattice distortion, as shown in Fig. 4c. The dislocations were not only present at the vicinity of GDC/BZY interface, but also extended through the GDC grains. The inversed fast Fourier transform (FFT) image in Fig. 4d clearly shows the existence of dislocations and lattice distortion by the compressive strain between BZY and GDC layers.

### Electrochemical Characterization

Figure 5 shows the polarization curves for μ-H-SOFCs using BZY electrolytes with and without the GDC cathode interlayer. Both fuel cells showed stable and high open-circuit voltages (OCVs) in the range of 0.98 to 1.07 V close to the theoretical thermodynamic value of 1.1 V, indicating that dense and pinhole-free electrolytes remained intact during the cell operation. The peak power densities obtained from the cell using BZY-only electrolyte were 51, 93 and 206 mW/cm^2^ at 350, 375, and 400 °C, respectively. For the cell with GDC interlayer, the peak power densities further increased up to 106, 187, 274 mW/cm^2^ at 350, 375, 400 °C, and reached 446 mW/cm^2^ at 425 °C. The peak power density values of both the cells with and without GDC interlayer were all higher than the reported values of μ-H-SOFCs at the same testing temperatures, as summarized in Table 1.

To confirm the improved reaction kinetics at the cathode side, the EIS curves of both the cells with and without the GDC interlayer were studied at 350 to 400 °C (Fig. 6). The first intercept on the real axis at high frequencies represents the Ohmic resistance R_Ω_, and the second intercept on the real axis at low frequencies corresponds to the total resistance of the cell^28^. For the polarization resistance R_p_, two distinguished arcs can be identified, where one has the characteristic frequency at the high frequency range (HF, ~10^5^ Hz) and the other at medium frequency range (MF, 10^2^–10^3^ Hz). The EIS curves were fitted using the equivalent circuit model of two parallel R and CPE (constant phase element) and one resistor connected in series, as shown in the inset of Fig. 6. The values extracted from the curve fitting were summarized in Table 2. The values of (R1, CPE1) and (R2, CPE2) corresponded to the HF and MF arcs, respectively. Each CPE has a CPE-T, which is related to the relaxation capacitance, and a CPE-P, which reflects the displacement of the center of the arc from the real axis^29^.

As summarized in Table 2, total polarization resistances R_p_ of the cell without GDC interlayer were 11.899, 7.045, and 3.660 Ωcm^2^ at 350, 400, and 450 °C, respectively, while the cell with GDC interlayer decreased to 4.136, 3.346, and 2.443 Ωcm^2^ at 350, 400, and 450 °C, respectively. The decrease in R_p_ indicates that the cathodic reaction was promoted by the additional GDC interlayer. Since the electrolyte and electrodes were identical for both cells across all experiments, the changes in R_p_ should be due to the presence of GDC interlayer. Ohmic resistances of the BZY electrolyte cell were 0.085, 0.089, and 0.099 Ωcm^2^, and for the GDC/BZY cell, the resistances increased slightly to 0.115, 0.129, and 0.131 Ωcm^2^ at 350, 375, and 400 °C, respectively, likely due to the additional thickness from GDC and the interface between GDC and BZY. The variations in the Ohmic resistance between these two cells can be negligible since they are relatively small as compared to the value of polarization resistance R_p_.

## Discussion

The improved cathode kinetics by the GDC interlayer can be identified from the corresponding Bode plot of each EIS curve (Fig. 6c). Two rate limiting steps were observed for both cells with and without GDC interlayer: the proton migration from the electrolyte to the TPBs, which corresponds to the HF resistance, and the oxygen dissociative adsorption and diffusion, which is related to the MF resistance^16^. With the GDC interlayer, MF resistances were decreased from 7.179, 4.582, and 2.557 Ωcm^2^ to 2.485, 2.070, and 1.776 Ωcm^2^ at 350, 375, and 400 °C, respectively, corresponding to the slightly depressed MF arc in the Bode plot with a frequency shift from 10^2^ Hz to 10^3^ Hz. The reduction in MF resistance means an enhancement in oxygen dissociative adsorption process on the GDC surface, which may originate from the high density of grain boundaries and dislocations in the GDC interlayer that provide preferential oxygen incorporation sites for lower interface resistance and faster surface exchange kinetics^30^,^31^,^32^.

**Figure 1:**
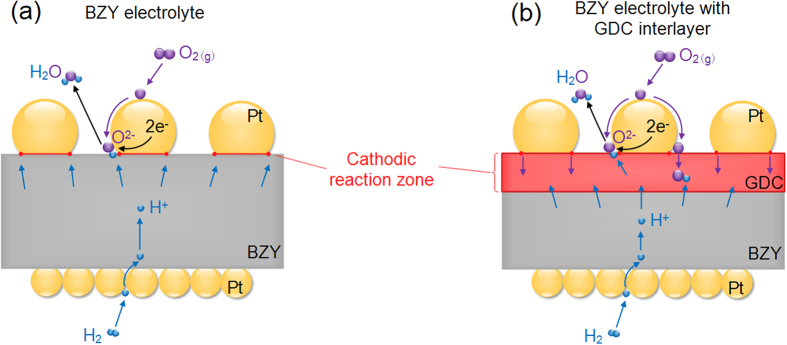
Schematics of μ-H-SOFCs with possible oxygen reduction reaction and water evolution reaction zone at the cathode/electrolyte interface. (**a**) The cell with platinum cathode in contact directly with BZY electrolyte, and (**b**) the cell with platinum cathode in contact with the GDC interlayer on top of the BZY electrolyte.

**Figure 2:**
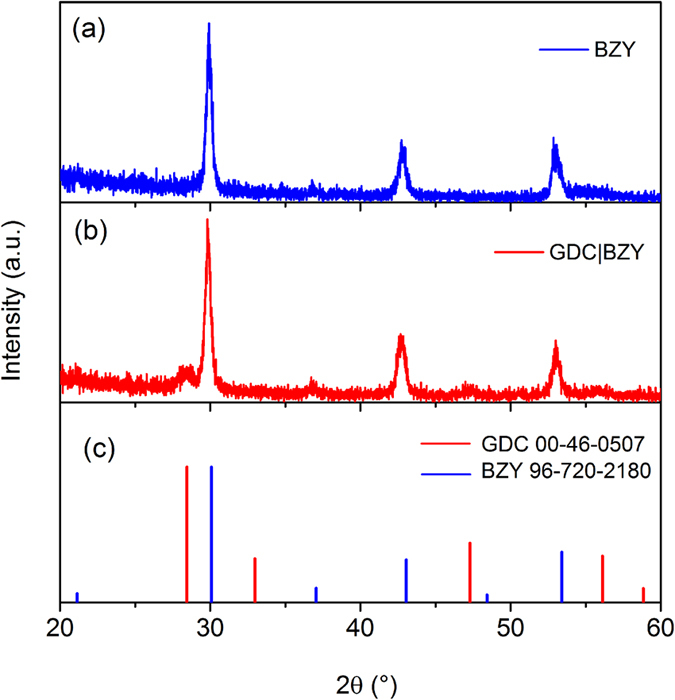
XRD patterns of BZY thin film electrolytes (**a**) with and (**b**) without GDC interlayer, with the reference peaks indices for BZY and GDC in (**c**).

**Figure 3:**
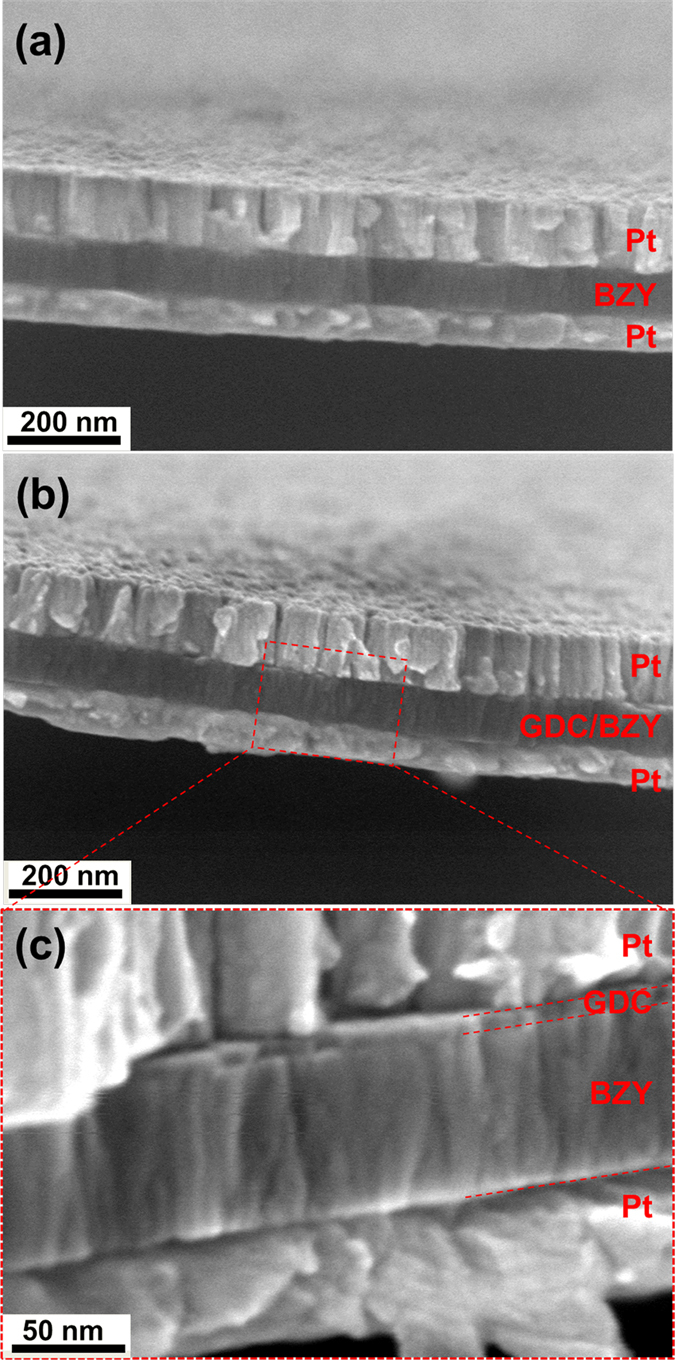
FESEM cross-sectional images of the free-standing electrolyte membranes with platinum cathode and anode. (**a**) BZY electrolyte; (**b**) BZY electrolyte with GDC cathodic interlayer, and (**c**) magnification of (**b**).

**Figure 4:**
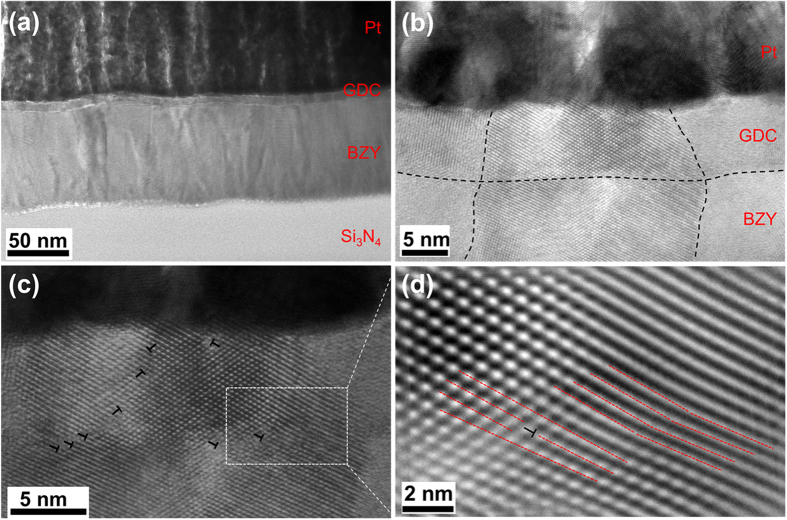
Cross-sectional TEM characterizations near the Pt/GDC-interlayer/BYZ-electrolyte at the non-freestanding region of the membrane on a Si_3_N_4_/Si substrate. (**a**) Cross-sectional view of the heterostructure of Pt/GDC/BZY/Si_3_N_4_; (**b**) High resolution TEM image of the GDC interlayer and interface. The GDC/BZY interface and grain boundaries are indicated by dashed lines; (**c**) A high density of dislocations is observed in the GDC interlayer, with some of them marked by the “⊥” labels; (**d**) The corresponding inverse FFT calculated image of the dotted region in (**c**). The dashed lines are guides indicating the dislocation lines and the existence of lattice distortion.

**Figure 5:**
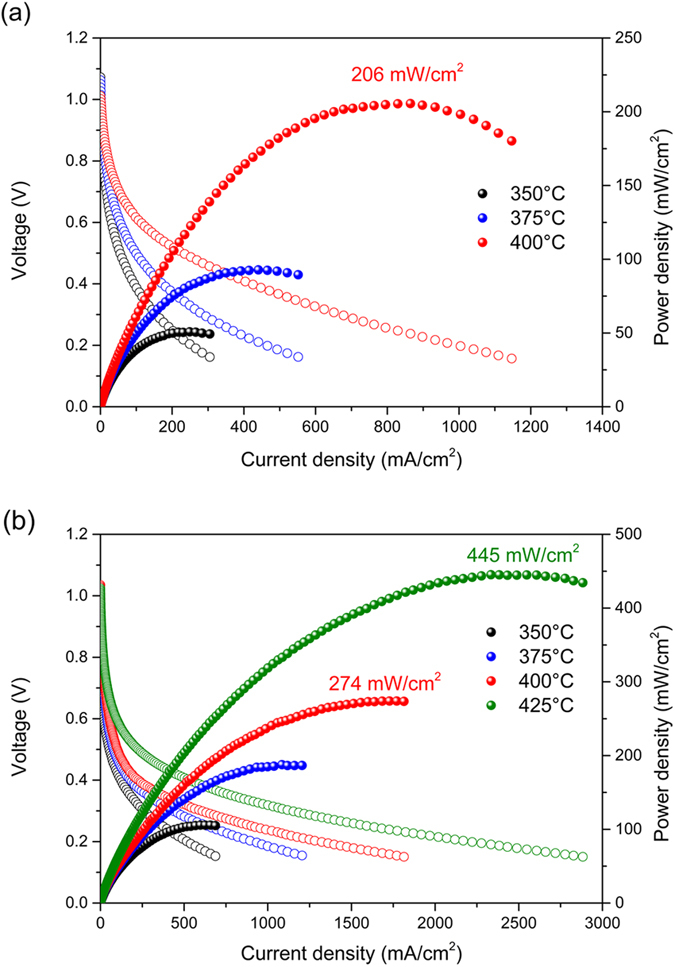
Current-voltage curves of the μ-H-SOFCs (**a**) with only BZY electrolyte, and (**b**) with BZY electrolyte with GDC interlayer at 350 to 425 °C.

**Figure 6:**
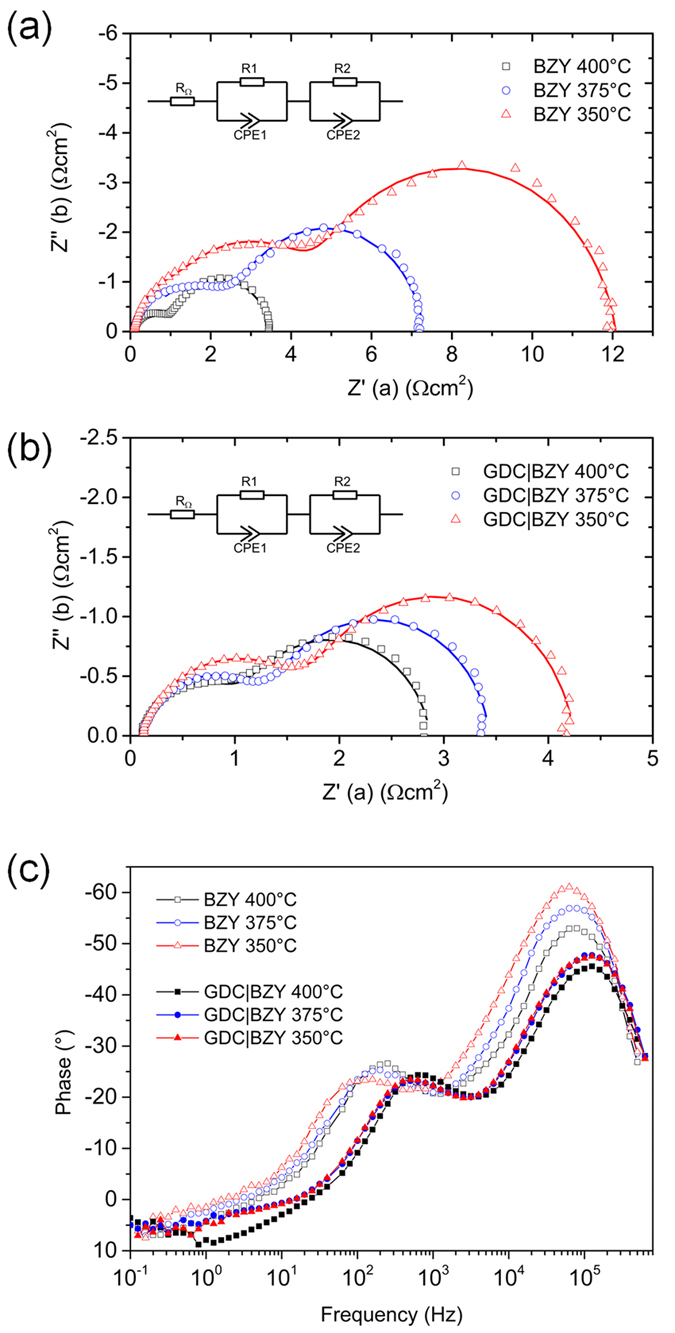
The Nyquist plots of EIS characterizations of μ-H-SOFCs with (**a**) BYZ electrolyte only, (**b**) BZY electrolyte with GDC interlayer, and (**c**) Bode plots for both cells.

The HF peaks in the Bode plot of the cell with GDC interlayer showed more apparent depression than that of the cell with only BZY electrolyte at all testing temperatures, indicating the enhancement of charge transfer process across the cathode/electrolyte interface in the presence of the GDC interlayer. The HF polarization resistances of the cell without GDC interlayer were 4.720, 2.463, and 1.103 Ωcm^2^ at 350, 400, and 450 °C, respectively, and the cell with GDC interlayer decreased to 1.651, 1.276, and 0.997 Ωcm^2^ at 350, 400, and 450 °C, respectively. The enhanced charge transfer process originated from the mixed conduction of proton and oxygen ion in the GDC layer, which can extend the reactions sites for water formation and evolution process. As depicted in Fig. 1b, the dissociative adsorbed oxygen ions can transfer from TPBs and surface grain boundaries to the GDC interlayer through oxygen vacancies. When proton reaches to the GDC/BZY interface, it can migrate to the GDC interlayer and react with the oxygen ions present within the GDC interlayer. Thus, the active regions involved in facilitating the water formation and evolution are not limited to the interface between Pt cathode and electrolyte, but extended to the GDC interlayer such that the HF resistance was decreased. Although the exact ORR and water evolution reaction mechanisms and pathways within the interlayer are still unclear, it is evident that the combined conduction of both O^2-^ and H^+^ has effectively improved the cathodic kinetics, leading to enhanced fuel cell performances at low temperature range for proton-conducting SOFCs.

In summary, the complex cathodic reactions in a μ-H-SOFC using BZY electrolyte were studied by an interface modification with the addition of an 8 nm-thick GDC cathode interlayer. The cathodic polarization resistance was effectively decreased by the additional GDC interlayer between the Pt cathode and BZY electrolyte. A record high peak power density of 445 mW/cm^2^ was obtained at 425 °C from the μ-H-SOFC with GDC/BZY bi-layer electrolyte. The EIS analysis of cathodic impedance of the fuel cells showed the enhanced cathodic charge transfer process across the cathode/electrolyte interface with the help of GDC interlayer, suggesting the effective promotion of proton and oxygen ion charge transfer as well as ORR and water evolution reaction between Pt cathode and BZY electrolyte through the GDC interlayer. The mixed oxygen and proton conduction in the GDC interlayer expanded the cathodic reaction sites from a 2-dimentional planar interface between Pt and BZY to nearly the entire GDC interlayer. The findings in this work show that cathodic interfacial resistance indeed has suppressed the possible high performance of μ-H-SOFCs. Further study on fundamental mechanisms into the protonic and oxygen ionic conduction pathways and reaction mechanism within the cathodic interlayer deserves an extensive exploration.

## Methods

### Electrolyte Deposition and Fuel Cell Fabrication

The μ-SOFCs are fabricated through a typical Si-based micro-machining process as previously reported^2^,^33^. A patterned Si_3_N_4_/Si with free-standing Si_3_N_4_ membrane (150 μm × 150 μm) was fabricated as the substrate for electrolyte deposition. BZY electrolyte and the GDC cathodic interlayer were both deposited by an ultra-high vacuum PLD/MBE system (PVD Products, USA) equipped with a 248 nm KrF excimer laser (Lambda Physik, Germany). Before the deposition, the chamber was evacuated to ultra-high vacuum of <10^−8^ Torr and then the free-standing substrates were heated up to desired deposition temperature of 800 °C with a heating rate of 25 °C/min. The distance between the target and substrate was kept at 80 mm. When the setting deposition temperature was reached, pure oxygen gas was introduced into the chamber to maintain a deposition pressure of 1 mTorr, which would ensure oxygen stoichiometry of the deposited sample. Sintered BaZr_0.8_Y_0.2_O_3−δ_ pellet target was ablated by a pulsed laser with a fluence of 3.0 J/cm^2^ at a repetition rate of 10 Hz for 30 minutes to fabricate BZY electrolyte thin film. The GDC cathodic interlayer was deposited subsequently by ablating a sintered Ce_0.9_Gd_0.1_O_1.95−δ_ for 4 minutes with the same deposition parameters. After thin film deposition, samples were cooled down to room temperature with a cooling rate of 25 °C/min. After the deposition of BZY electrolyte and GDC interlayer by PLD, the Si_3_N_4_ supporting layer was removed by relative ion etching (RIE) with CF_4_ gas, resulting in a free-standing nanoscale electrolyte. Porous platinum thin films are deposited on both sides of the electrolyte via RF sputtering technique with Ar pressure of 30 mTorr at room temperature to achieve porous anode and cathode.

### Thin Film Crystallinity and Morphology Characterization

The crystallinity and structural phase of deposited films were analyzed by grazing incidence X-ray diffraction (GIXRD) system equipped with a CuKα X-ray source (PANalytical Empyrean XRD, Netherlands) operating at beam intensity of 40 kV and 40 mA. The glancing angle X-ray was incident at an angle of 0.5°. Cross-sectional micro-structure of the fuel cell was characterized by field-emission scanning electron microscopy (FESEM, JSM-7600F, JEOL, Ltd., Japan) and transmission electron microscopy (TEM, JEM-2100, JEOL, Ltd., Japan).

### Fuel Cell Performance Characterization

Fuel cell performance was tested with a customized test station for the measurement of silicon-based μ-SOFCs. The μ-SOFC chip was fixed on a stainless steel chamber with gold gasket for sealing. The furnace temperature was elevated to the set testing temperature with a heating rate of 10 °C/min. An Au coated tungsten probe, which was connected to a micro-positioner, was put in contact with the porous platinum cathode for current collection. Dry hydrogen fuel with a flow rate of 20 sccm was supplied to the anode side, and the cathode side was exposed to the ambient air. The fuel cell performance was measured by obtaining the current-voltage polarization curves at temperatures from 350 °C to 425 °C. For data collection of both the polarization curves and EIS spectra, a Solartron 1470E potentiostats system and a 1255B Frequency Response Analyzer (FRA) were connected to the anode and cathode sides. The obtained EIS curves were fitted with equivalent circuit models using Zview software (Scribner Associates).

## Additional Information

**How to cite this article**: Li, Y. *et al.* Proton-conducting Micro-solid Oxide Fuel Cells with Improved Cathode Reactions by a Nanoscale Thin Film Gadolinium-doped Ceria Interlayer. *Sci. Rep.* **6**, 22369; doi: 10.1038/srep22369 (2016).

## Figures and Tables

**Table 1:** Summary of μ-SOFCs performances with protonic ceramic electrolytes reported in the literature.

Group	Reference	Cell Structure	Materials (anode-electrolyte-cathode)	Electrolyte Thickness (nm)	OCV (V)	Peak Power Density (mW/cm^2^)	Temperature (°C)	Fuel
Nanyang Technological University	This work	Free-standing	Pt-BZY-Pt	75	1.02	206	400	H_2_
			Pt-BZY/GDC-Pt	83	1.04	274	400	H_2_
					1.03	446	425	H_2_
	Su *et al.*^7^	Free-standing	Pt-BZY-Pt	300	0.56	8	400	H_2_
	Ha *et al.*^34^	AAO supported	Pt-BZY-Pt	900	0.8	6	250	CH_4_O
	Li *et al.*^33^	Free-standing	Pt-BCY-Pt	300	0.59	30	400	H_2_
Stanford University	Shim *et al.*^35^	Free-standing	Pt-BZY(PLD)-Pt	130	1.12	120	450	H_2_
			Pt-BZY(ALD)-Pt	110	1.09	136	400	H_2_
	Kim *et al.*^11^	Free-standing	Pt-BZY-Pt	120	0.85	186	450	H_2_
Korea University	Bae *et al.*^10^	Free-standing	Pt-BCY-Pt	200	0.98	145	400	H_2_
			Pt-BCY/BZY-Pt	200	0.89	48	400	H_2_
			Pt-BZY/BCY/BZY-Pt	200	0.78	8	400	H_2_
			Pt-BZY-Pt	200	1.08	27	400	H_2_
			Pt-BZY/BCY-Pt	200	1.06	40	400	H_2_
			Pt-BCY/BZY/BCY-Pt	200	1.05	62	400	H_2_
Seoul National University	Kang *et al.*^36^	AAO supported	Pd-BZY-Pt	1000	1.0	9	400	H_2_
	Chang *et al.*^37^	AAO supported	Pt-BZY-Pt	1000	1.04	44	450	H_2_
	Park *et al.*^38^	AAO supported	Pt-BZY-Pt	1340	1.1	21	450	H_2_
Harvard University	Adam *et al.*^39^	Free-standing	Pt/Pd-BZY-Pt	136	0.95	40	495	H_2_

BCY: Y-doped BaCeO_3_

ALD: Atomic layer deposition.

**Table 2:** Summary of the values extracted from equivalent circuit fitting of the EIS curves at 350, 375, and 400 °C.

Fuel Cell Electrolyte	Temperature (°C)	R_Ω_ (Ωcm^2^)	R_p_ = R_1_ + R_2_(Ωcm^2^)	HF Semicircle			MF Semicircle		
				R_1_ (Ωcm^2^)	CPE1-T (F)	CPE1-P	R_2_ (Ωcm^2^)	CPE2-T (F)	CPE2-P
BZY	350	0.099	11.899	4.72	7.09E-09	0.872	7.179	2.24E-07	0.779
	375	0.089	7.045	2.463	8.33E-09	0.864	4.582	2.18E-07	0.818
	400	0.085	3.660	1.103	9.06E-09	0.869	2.557	2.07E-07	0.855
GDC/BZY	350	0.131	4.136	1.651	1.57E-08	0.789	2.485	9.83E-08	0.928
	375	0.129	3.346	1.276	1.33E-08	0.809	2.07	8.66E-08	0.933
	400	0.115	2.443	0.997	1.27E-08	0.82	1.776	7.70E-08	0.902
